# Food and Drug Adulteration. Their Medical and Legal Significance1Read before the Society of Medical Jurisprudence, May 11, 1908.

**Published:** 1908-10

**Authors:** Thomas Darlington

**Affiliations:** Commissioner of Health. New York


### Food and Drug Adulteration. Their Medical and Legal Significance.[1]

By THOMAS DARLINGTON, M. D.
Commissioner of Health. New York.
(*Medical Review of Reviews*, August 1908.)

THE term "adulteration" when applied to food or drugs is broad in its scope, but has been given a well-defined legal meaning. In general it may be said that adulteration consists in subtracting from a substance any part of its natural or inherent qualities or adding to it any ingredient which would render

1. Read before the Society of Medical Jurisprudence, May 11, 1908.

the article of a different nature than that of its expressed and natural form. This may be intentional or accidental, and the latter term covers the large class of permissible adulterants, such as the addition to foodstuffs of substances having themselves a food value.

Salt, sugar, vinegar and spices come within this class. Smoke may be used as a preservative of meat, and starch is commonly added to the salts composing baking powder for the purpose of absorbing moisture. In other words, an attempt at deceit must be considered.

Almost coexistent with the growth of this wonderful country, and seemingly as a part of the fierce business competition which has insidiously developed, this evil of food and drug adulteration has arisen. Individual states have in the past made laws which dealt more or less vigorously with the conditions which come within their jurisdiction. These efforts at reform, however, have been spasmodic in character and widely differing in their nature. Commendable though they were, their reformatory effect has been necessarily local and their execution, in many instances, scarcely more than admonitory, not prohibitive.

The conditions of substitution and adulteration of foods and drugs were little short of deplorable less than a decade ago, and no half-hearted measures could be relied upon to deal with this blot on the face of our boasted civilisation and high standards of right living. Probably no other form of wrongdoing could so intimately touch each one of us as this tampering with our food and medicines, yet our sluggish national conscience seemed a long time in arousing itself to deal with this patent yet insidious evil. Notwithstanding the positive assertion from certain mercenary quarters that they, not we, were to be the best judges of what was good for us, our national lawmakers, backed by the aroused sense of wrong on the part of their constituents, and in response to an urgent public demand, formed and passed what may well be called one of the most far-reaching and radical laws of modern times, the so-called "Pure Food Law."

Possibly you are all more or less acquainted with the component parts of this piece of beneficent legislation, but it is so direct in its statements and so inclusive in its meaning that I wish to repeat its exact definitions of adulteration and that other description, "misbranding."

In Section 7 of this act, which in its entirety was approved on June 30, 1906, it is stated "that for the purpose of this act an article shall be deemed to be adulterated: In case of drugs:

"*First*—If when a drug is sold under or by a name recognised in the United States Pharmacopeia or National Formulary it differs from the standard of strength, quality or purity as determined by the test laid down in the United States Pharmacopeia or National Formulary official at the time of investigation. Provided that no drug defined in the United States Pharmacopeia or National Formulary shall be deemed to be adulterated under this provision if the standard of strength, quality or purity be plainly stated upon the bottle, box or other container thereof, although the standard may differ from that determined by the test laid down in the United States Pharmacopeia or National Formulary.

"*Second*—If its strength or purity fall below the professed standard or quality under which it is sold.

"In the case of food:

"*First*—If any substance has been mixed or packed with it so as to reduce or lower or injuriously affect its quality or strength.

"*Second*—If any substance has been substituted wholly or in part for the article.

"*Third*—If any valuable constituent of the article has been wholly or in part abstracted.

"*Fourth*—If it be mixed, colored, powdered, coated or stained in a manner whereby damage or inferiority is concealed.

"*Fifth*—If it contain any added poisonous or other added deleterious ingredient which may render such article injurious to health; provided that, when in the preparation of food products for shipment they are preserved by any external application applied in such manner that the preservation is necessarily removed mechanically, or by maceration, in water, or otherwise, and directions for the removal of said preservative shall be printed on the covering or the package, the provisions of this act shall be construed as applying only when said products are ready for consumption.

"*Sixth*—If it consists in whole or in part of a filthy, decomposed or putrid animal or vegetable substance, or any portion of an animal unfit for food, whether manufactured or not, or if it is the product of a diseased animal, or one that has died otherwise than by slaughter."

Section 8 of this act, which deals with the subject of misbranding, is necessarily somewhat long, and I will not read its full provisions. Its most important points are well covered in that part which states: "That the term 'misbranded' as used therein shall apply to all drugs or articles of food, or articles which enter into the composition of food, the package or label of which shall bear any statement, design or device regarding such article, or the ingredients or substances contained therein which shall be false or misleading in any particular, and to any food or drug product which is falsely branded as to the State, Territory or country in which it is manufactured or produced." One other provision is of sufficient importance to be mentioned; that is, the section which states that "all packages containing food or drugs must have stated on the label the quantity or portion of alcohol, morphine, opium, cocaine, heroin, alpha or beta eucaine, chloroform, cannabis indica, chloral hydrate or acetanilid or any derivative or preparation of any such substances which may be contained therein."

This law in its entirety is epoch-marking. It covers all interstate traffic and transportation, and must well be effective in restraining cupidity when practised on any extensive scale.

In addition to its provisions, there are in every community and state certain local conditions of this nature which may be dealt with only by the local authorities, for the production and sale within its own borders of any articles of the above-mentioned classes can only be met by the action of the governing power of the locality affected. At the present time the pure-food laws of many states and cities are almost entirely similar to the national laws. So that, in addition to the Federal officials who are charged with the enforcement of the national laws, we have here in New York city the inspectors of the Department of Health, who are constantly on the alert to detect violations of those sections of the Sanitary Code which deal with conditions of a like nature. Sections 68 and 69 of the Code are very similar in their phraseology with the relative sections of the Federal laws, and in the Board of Health of this city is vested the authority to deal with the local production and sale of these products.

Two questions must be considered in dealing with this matter: first, the intent to deceive or the element of fraud, and, second, the influence of the adulteration upon our health.

All misbranding and all adulteration of food and drugs, unless the fact of the adulteration is stated, is deception. The adulterant may be harmless, but a person has the right to know what he is buying, and if there be any comparative values in right we must assume that it is of the highest type when the question of the food we eat is involved.

In the line of harmless adulteration may be mentioned the sale of skimmed milk. That such milk has a definite food value is undeniable. If its sale in its proper guise could be assured, it would fill a well-defined place in the list of food-stuffs. The real harm, however, comes in the fact that it would require almost superhuman effort to prevent its being sold as whole milk. Our sanitary code requires that milk shall contain 3 per cent. of butter fats. If this fat in the shape of cream has been removed from the milk, the sale of the remainder is a fraud and an imposition upon the consumer. So the addition of water to milk, harmless though it may be, as far as deleterious effects are concerned, is a deception.

The addition of preservatives to milk is a different matter. Formaldehyde seems to be the favorite in this regard. Used in the usual quantity—one teaspoonful to a 40-quart can of milk—its actual harmful effect upon health may be debatable. The main harm arises because the preservative allows the dealer to sell milk a week old under the assumption that it is fresh. The taste or the odor of the formaldehyde in such proportions is not distinguishable; its presence can be detected only by means of chemical tests. If used in larger quantities, or if used in the smaller amount for long-continued periods of time, we may well question whether it will not produce gastrointestinal disturbances.

Somewhat the same conditions exist in regard to the preservatives used to keep meat fresh or to treat putrid meat so that it may appear attractive to the eye and pleasant to the palate. Here it is not a question of stating on a label attached to the meat the fact that a preservative has been used. The main ingredient in these meat preservatives is sodium sulphite, a salt which has its place as a medicinal agent, but which some investigations seem to show is harmful to health when used for any long period of time. Whether this be so or not, a large part of the usefulness of these meat preservatives has been in their use upon chopped meat, decomposed or putrid, thereby restoring it to a salable condition without restoring its wholesome qualities. Such deception is criminal.

Coloring or coating food products so that they may have an attractive appearance is a form of deception which has been largely practised. Preserved fruits or vegetables may have a good, bright color, but never have the brilliant reds and greens so often observed in the artificially-colored products. While analine dyes, which are used to produce the reds of the berries and fruits, and copper sulphate, which gives the intense greens sometimes found in canned peas and beans, may not be actively harmful to health, their use cannot be condoned unless the buyer prefers to satisfy his esthetic preferences at the expense of his inner man. Benzoic acid and the benzoates, salicylic acid and borax, are the preservatives most commonly used in canned foodstuffs. Elaborate, detailed and painstaking experiments as to the results upon the human body of long-continued use of these substances have been undertaken under the direction of Dr. H. W. Wiley, chief chemist of the United States Department of Agriculture. In a paper on "The Influence of Preservatives and Coloring Matters and Their Relation to Nutrition" read before the New York Medical Association in October, 1905, Dr. Wiley has summed up his conclusions in regard to the matter in the following words: "The experiments which go to show that no hurt has come from the use of preservatives and coloring matters are in reality condemnatory. Their use must be justified by positively beneficial results. On the contrary, with the prejudicial results obtained, the use of preservatives and coloring matters in foods, even in the small percentage of cases in which they are used, is or should be condemned. My own experience leads me to believe that the use of even small quantities of ordinary preservatives for a long while or large quantities for a short while leads to unfavorable effects. There is a general loss of weight, a feeling of lassitude and malaise, and in some cases more pronounced symptoms of intestinal and metabolic disturbances. If it be assumed that with wholesome food the metabolic relations which are observed are normal, and these relations are changed on the addition of preservatives and coloring matters, then this change, no difference what it may be, must be regarded as prejudicial, because it is not normal."

Since the approval of the national food law and the enactment of the similar provisions in the sanitary code of the Board of Health, which were approved on September 10, 1906, there has been a widespread and radical reform in regard to these matters within New York city. The Board of Health is working in harmony with the Federal authorities, and our experience coincides with that of the Secretary of Agriculture, which he expressed on December 1, 1906, in the following words:

"From the tenor of the correspondence received at this department, and from the oral hearings which have been held, it is evident that an overwhelming majority of the manufacturers, jobbers and dealers of the country are determined to do their utmost to conform to the provisions of this act, to support it in every particular and to accede to the opinions of this department respecting its construction."

Personally, I have had many conferences with the manufacturers and dealers of this city, and the same spirit of willingness to conform with the laws has been manifested on their part.

Our inspectors are constantly on the alert to detect infringement of the laws. Samples of food which may be suspected of adulteration or misbranding are sent to the chemical laboratory of the department and there subjected to analysis. Misbranding may often be detected by mere inspection and investigation. Small fish caught off our coast, packed in oil and labeled "Imported Sardines" may be pure, attractive and satisfying to our sense of taste, but the practice cannot be defended on moral grounds, and a large variety of our supposedly foreign delicacies have been unable to withstand a critical investigation of their origin.

A subject which has merited considerable attention on our part has been the large traffic which is carried on in so-called "spot" and "canned" eggs. This practice, which is thoroughly reprehensible, consists of setting aside those eggs which show a spot when held to a light. They are not fit for the fresh-egg trade, so are broken, the contents emptied into cans, a proportion of some preservative, usually formaldehyde, is added and the cans sealed. Packed in this manner the eggs are preserved for a long period of time. Their sale is mainly to manufacturers of pies, cakes and biscuits. The fact that the eggs are themselves in a condition unfit for human consumption, added to the fraud involved in covering up their deficiencies with a preservative which hides their true state, reveals a double iniquity. Large consignments of these eggs have been seized and destroyed, and the department is at the present time confronted with a suit for damages involving some $2,000, the amount claimed as representing the assumed value of one seizure of these eggs.

It is my desire to proceed along the lines of the enforcement of this law in complete harmony and unity with the interests involved, and unwarranted interference with the rights of property or business holds no place in our plans, but evasion or studied non-compliance with the law merits and will receive stringent and forcible attention.

From a legal point of view, canned goods present a rather intricate question. It is manifestly impossible for an inspector or chemist to swear that because one can of a certain brand has been found to contain a preservative not noted on the label all other cans of the same brand likewise contain the same preservative. Condemnation and seizure of the entire stock of a dealer on such grounds would not stand the test of law, but the remedy consists in bringing suit on the basis of the one can whose contents were proved to be adulterated. This procedure is usually effective in forcing the retirement of the whole consignment.

It is not my purpose to take up in detail the specific action on health of each form of preservative. To do so would be simply a reiteration of established facts and a repetition of certain primary dictums of therapeutics; nor, at the risk of a tiresome and too lengthy discourse, may I discuss the full procedure used by the Department of Health in dealing with this class of cases. I would, however, mention briefly our system of meat inspection which follows up the animals intended for slaughter and food consumption from the time of their arrival within the city limits through the process of slaughtering the animal, dressing the meat and its transportation through the streets, until it passes through the retailer's hands and is sold to the consumer. During the last six months of 1907, as a part of this work, 41,824 inspections were made of slaughter houses, stockyards, butcher shops, packing-houses and other places connected with the meat industry; during the year 2,974,948 pounds of meat were condemned and destroyed.

The city markets, retail stores, bakeries and other places where food is prepared and sold, and even the pushcarts, are constantly under our supervision. During the past year 3,533,-858 pounds of such foodstuffs, including fish, were seized and destroyed, while 7,540 516 pounds of fruits met the same fate. This immense quantity of edibles was unfit for food, some part of it by reason of the addition of foreign substances and some because of its natural decay. In a number of specimens of food analyzed to determine the presence and character of suspected preservatives 16 instances were found in which chopped meat, or so-called Hamburger steak, contained sulphurous acid. Smoked liverwurst was preserved with both sulphurous and boric acids; frankfurters found both artificially colored and containing boric acid; vinegar doctored with sulphuric acid, and "olive oil" made up largely of cottonseed oil; gelatine containing sulphites; "lemon extract" with no lemon in it, and "pure honey" found to be glucose with a small quantity of sugar.

On the whole the analyzed products have conformed with the letter of the law, and I have simply cited the above examples to show that there is still need of vigilance. I believe that the quality of the food now sold in this city maintains a high average, and I do not wish to be understood as sounding any note of alarm regarding its purity nor the honesty of our merchants.

Although these violations of the law have been brought to the attention of the Corporation Counsel, and actions have been brought by him against the offending parties, as yet no cases have been taken upon appeal, and consequently there are no written opinions so far as the Department of Health is concerned in cases handled by it. A case under the state law, which is somewhat similar to our sanitary code, has just been decided in regard to a brand of tomato catsup which was labeled as follows: "Prepared from whole ripe tomatoes; no artificial color, and contains one-tenth of 1 per cent. of soda benzoate." This catsup was found to contain double the stated quantity of soda benzoate, a clear case of misbranding, and held to be a violation of the law.

Before the insertion in the sanitary code of those sections which have to do with the adulteration and misbranding of food and drugs the Department of Health had proceeded against the evil of drug substitution and deceptive branding of medicines. Section 65 of the code gave ample authority for this work, and numerous instances were discovered of direct substitution of drugs in physicians' prescriptions, as well as that other fraud, the careless or intentional difference in the quantity of the drug dispensed from the quantity of drug ordered. In the former class the commonest forms of substitution were the use of acetanilid in place of the then more expensive phenacetine, and the use of wood alcohol where grain alcohol was indicated. In the second class the evil was even more widespread. Instances were not uncommon where a prescription called for a number of capsules or powders, each containing a definite amount of a drug or a mixture of drugs, the dispensed packages were each found to contain from one-half to three-quarters of the quantity called for. Crude drugs, extracts and tinctures which were stated as of a definite pharmaceutical strength in the United States Pharmacopeia were ordered, and the dispensed article found to be made up of drugs assaying only a fraction of the standard of total alkaloids.

If substitution in the case of food may be regarded as an evil, substitution in the case of drugs reveals such a greater degree of iniquity that it can only be regarded as a crime. Persons in health and the full degree of an alert mentality may to a great degree protect themselves against fraud and deception, but the helpless invalid should receive tender consideration and undeviating honesty from those persons in whom he has placed faith to the extent of trusting his regained health, or even his life itself. Many a conscientious physician, trusting in the known efficacy of a drug of known potency and power, has been puzzled and disheartened when following its administration the expected result has not ensued. Here it is not a question of a slight deviation in the standard of normal health. Prolonged illness or even death may be the outcome of this reprehensible fraud on occasions. The retail druggist may be entirely guiltless and the fault may be traced to the manufacturer.

In analysis of 29 samples of crude drugs for their alkaloidal strength made by the Department of Health not one was found to conform to the standard. Samples of powdered belladonna leaves with a standard of .35 per cent. of total alkaloids were found to contain amounts varying from .06 to .21 per cent. Powdered opium varied in its percentage of morphine from 11 to 14 per cent., while a sample of saffron examined showed 28 per cent. fine saffron petals and 72 per cent. analine-dyed petals, probably magnolia or daisy. That crude drugs may naturally vary in these degrees is well known. The fraud lies in the concealment of the true state of affairs, and the remedy in their assaying and the plain labeling of their alkaloidal strength.

In the endeavor to correct these conditions we have had the earnest co-operation of nearly all the large firms of druggists, and such a state of affairs no longer exists.

Misbranding of drugs and medicinal mixtures covers a multitude of sins. The opium, cocaine and whisky habits often date their onset from the use of patent medicines containing them. The moral and mental degradation of the victims and the resultant and widespread suffering entailed as a result of this vicious taste is too well known to need more than a passing mention. The cocaine habit alone has grown to alarming proportions. Its sale, other than on the prescription of a physician, is now prohibited, and I am glad to record that one of the most notorious offenders in this respect has had the opportunity of repenting of his misdeeds afforded by an enforced six months' imprisonment.

Catarrh powders, under many designations, have been found to owe their soothing effects and long-continued use to the presence of this drug, while mixtures for internal use and variously labeled as "tonics" and "pain killers" have long owed their popularity to the soothing effect of the opium or the exhilaration of the whiskey they contained.

Substitution, adulteration and misbranding are potent frauds, but the American people may always be trusted to restore the balance of right. The great moral awakening which has made possible the passage and enforcement of "the pure food and drug law" has also shown itself in the general co-operation on the part of the producer and dealer. The millennium may not yet be in sight, but conditions are rapidly improving, and the future is bright with promise. Constant vigilance may still be necessary, but the law is becoming less and less difficult of enforcement, and the final recognition of its just and beneficent purpose is well within the realm of possible attainment.

